# Treatment-Free Survival After Nivolumab vs Pembrolizumab vs Nivolumab-Ipilimumab for Advanced Melanoma

**DOI:** 10.1001/jamanetworkopen.2023.19607

**Published:** 2023-06-23

**Authors:** Mehul Gupta, Igor Stukalin, Daniel Meyers, Sid Goutam, Daniel Y. C. Heng, Tina Cheng, Jose Monzon, Vishal Navani

**Affiliations:** 1 Division of Medical Oncology, Department of Oncology, Tom Baker Cancer Centre, Calgary, Alberta, Canada; 2 Faculty of Medicine and Dentistry, University of Alberta, Edmonton, Alberta, Canada

## Abstract

**Importance:**

Treatment-free survival (TFS) represents an alternative time-to-event end point, accurately characterizing time spent free of systemic therapy, providing a more patient-centric view of immune checkpoint inhibitor (ICI) therapy regimens. There remains a lack of studies evaluating TFS outcomes among patients with advanced melanoma who are receiving immunotherapy, especially outside of the clinical trial setting.

**Objective:**

To evaluate TFS outcomes for patients with advanced melanoma receiving first-line ICI therapy outside of a clinical trial setting.

**Design, Setting, and Participants:**

This multicenter cohort study of patients with advanced melanoma receiving first-line ICI therapy between August 1, 2013, and May 31, 2020, was conducted in Alberta, Canada. Data analysis was performed in August 2022.

**Exposures:**

Patients received standard-of-care, first-line ICI therapy treatment regimens including single-agent nivolumab, single-agent pembrolizumab, or ipilimumab-nivolumab.

**Main Outcomes and Measures:**

Treatment-free survival was defined as the difference in the 36-month restricted mean survival time between 2 conventional survival end points: (1) time from treatment initiation to ICI cessation, death, or censoring at last follow-up and (2) time from treatment initiation to subsequent systemic anticancer therapy, death, or censoring at last follow-up.

**Results:**

A total of 316 patients with advanced melanoma receiving first-line nivolumab (n = 51; median age, 66 years [IQR, 56-78 years]; 31 men [60.8%]), pembrolizumab (n = 158; median age, 69 years [IQR, 60-78 years]; 112 men [70.9%]), or combination nivolumab-ipilimumab (n = 107; median age, 53 years [IQR, 42-60 years]; 72 men [67.3%]) were included. Treatment groups were similar with regard to sex, primary tumor location, and presence of metastasis, although patients receiving combination nivolumab-ipilimumab had a lower Eastern Cooperative Oncology Group status, were younger, and were more likely to be *BRAF* V600E positive than those receiving anti–programmed cell death protein 1 (anti–PD-1) monotherapy. The restricted mean TFS was longer for nivolumab-ipilimumab (12.4 months [95% CI, 8.8-16.0 months]) compared with nivolumab (8.9 months [95% CI, 4.4-13.5 months]) and pembrolizumab (11.1 months [95% CI, 8.5-13.8 months]). During the 36-month follow-up interval, patients treated with nivolumab-ipilimumab spent 34.4% of their time (12.4 of 36 months) not receiving systemic anticancer treatments compared with 30.8% (11.1 of 36 months) and 24.7% (8.9 of 36 months) of the time for the pembrolizumab and nivolumab treatment groups, respectively.

**Conclusions and Relevance:**

This cohort study of patients with advanced melanoma receiving first-line ICI therapy suggests that TFS represents a patient-centric, informative end point. Patients treated with combination nivolumab-ipilimumab spent more time alive and free from systemic anticancer therapy than those treated with anti–PD-1 monotherapy alone.

## Key Points

**Question:**

How do treatment-free survival outcomes differ between various first-line immune checkpoint inhibitor therapy regimens for patients with advanced melanoma?

**Findings:**

In a muticenter cohort study of 316 patients with advanced melanoma treated with first-line immune checkpoint inhibitor therapy, patients treated with nivolumab-ipilimumab experienced a mean treatment-free survival of 12.4 months compared with a mean of 8.9 months for single-agent nivolumab and 11.1 months for single-agent pembrolizumab.

**Meaning:**

This study suggests that patients with advanced melanoma treated with first-line nivolumab-ipilimumab experienced numerically longer treatment-free survival compared with single-agent nivolumab or pembrolizumab.

## Teaser

This cohort study evaluates treatment-free survival outcomes for patients with advanced melanoma receiving first-line immune checkpoint inhibitor therapy outside of a clinical trial setting.

## Introduction

The advent of immune checkpoint inhibitors (ICIs) has led to a fundamental shift in the approach to treatment of advanced melanoma, resulting in significant improvement in survival outcomes for these patients.^1,2^ In particular, anti–programmed cell death protein 1 (anti–PD-1) monotherapies with agents including pembrolizumab or nivolumab are established regimens for advanced melanoma management, as are combination therapies consisting of anti–PD-1 agents and the cytotoxic T-cell lymphocyte antigen 4 blockade-agent ipilimumab.^3^

Analyses of the effectiveness of these treatment regimens have largely relied on conventional time-to-event outcomes, such as overall survival.^4,5,6^ Although quite useful to assess survival benefit, these measures often fail to capture other important, patient-oriented aspects of care. Patients who discontinue ICI therapy due to toxic effects or prolonged duration of disease control are able to receive the ongoing benefit from these agents as they are able to remain free of subsequent systemic anticancer (SSAC) therapy. A better understanding of this dimension of care is crucial because multiple studies have found an associaton between increased time spent free of SSAC and improvements in patient quality of life.^7,8^ Treatment-free survival (TFS) quantifies time spent free of SSAC therapy by measuring the period between cessation of first-line treatment and initiation of subsequent lines of therapy. Treatment-free survival has been examined among patients with advanced melanoma receiving first-line ICI therapy in the clinical trial setting^9,10^; however, an examination of TFS in a routine care setting for this patient population has yet to be reported, to our knowledge. A more thorough understanding of the TFS outcomes experienced by patients with advanced melanoma treated with first-line ICI therapy may help inform therapeutic decision-making, given the increasing number of treatment choices available to clinicians and patients in this setting.

## Methods

### Patient Selection

Patients were selected from the Alberta Immunotherapy Database (AID) study, which is a multicenter observational cohort study being conducted in Alberta, Canada. The AID study retrospectively captured demographic, clinical, pathologic, and outcome data for patients having received ICI therapy between August 1, 2013, and May 31, 2020. Of the patients included in the AID cohort, those selected for this cohort study were required to have a histologically confirmed, locally advanced form of metastatic melanoma (stage III unresectable or stage IV metastatic melanoma), agnostic of primary site of origin, as well as to have received at least 1 cycle of first-line ICI therapy with either single-agent nivolumab, single-agent pembrolizumab, or combination nivolumab-ipilimumab. This study was reviewed and approved by the health research ethics board of the Alberta–Cancer Committee, which waived patient consent given the low-risk, deidentified, retrospective nature of this work. This study followed the Strengthening the Reporting of Observational Studies in Epidemiology (STROBE) reporting guideline.

### Statistical Analysis

Statistical analysis was conducted in August 2022. Treatment-free survival was calculated for each ICI therapy subgroup of patients with advanced melanoma and defined as the area under the curve between 2 time-to-event end points: time to ICI therapy discontinuation and time to subsequent SSAC therapy initiation. Time to ICI therapy discontinuation was defined as the number of months between ICI therapy initiation and the first of therapy discontinuation for any reason, death, or censoring at last known time alive. Time to SSAC therapy initiation was defined as the number of months from ICI therapy initiation to the first of SSAC therapy initiation, death, or censoring at last known time alive. The difference between these 2 time-to-event outcomes represents the time that patients were able to spend free of SSAC therapy and was determined using a restricted mean survival time approach at 36 months. This time point was selected given that the median follow-up of included patients in this study was 36.1 months (IQR, 26.1-43.8 months), as assessed by the reverse Kaplan-Meier method.^11^ Time-to-event end points were evaluated using the Kaplan-Meier method. Enhanced swimmer plots were developed to visualize TFS outcomes at 36 months.^12^ All statistical analyses were carried out in R, version 4.0.2 (R Group for Statistical Computing). All statistical tests were 2-sided, and *P* < .05 was considered to indicate statistical significance. No formal statistical testing was undertaken due to the observational nature of this work, and all results are presented in a descriptive manner alone.

## Results

A total of 316 patients were included in this analysis, including patients receiving single-agent pembrolizumab (n = 158; median age, 69 years [IQR, 60-78 years]; 112 men [70.9%]), single-agent nivolumab (n = 51; median age, 66 years [IQR, 56-78 years]; 31 men [60.8%]), or combination ipilimumab-nivolumab (n = 107; median age, 53 years [IQR, 42-60 years]; 72 men [67.3%]). Baseline participant characteristics at the time of ICI therapy initiation as stratified by therapy received are shown in the Table. Cohorts were similar with regard to patient sex, primary melanoma site, presence of liver metastases, and lactate dehydrogenase level. The cohorts differed significantly with regard to median age, Eastern Cooperative Oncology Group (ECOG) status (ECOG <1: nivolumab, 19 of 51 [37.3%]; pembrolizumab, 61 of 157 [38.9%]; ipilimumab-nivolumab, 63 of 107 [58.9%]), clinical stage (stage IV: nivolumab, 42 of 51 [82.4%]; pembrolizumab, 137 of 158 [86.7%]; ipilimumab-nivolumab, 86 of 107 [80.4%]), and *BRAF* (OMIM 164757) V600E variant status (*BRAF* variant present: nivolumab, 2 of 51 [3.9%]; pembrolizumab, 19 of 158 [12.0%]; ipilimumab-nivolumab, 32 of 107 [29.9%]). ECOG status represents an individual’s level of function with regard to their ability to care for themselves and perform daily activities rated on a scale from 0, which indicates no restrictions, to 5, which is death.

**Table.** Baseline Clinical, Pathologic, and Laboratory Variables of Patients With Advanced Melanoma Stratified by Immune Checkpoint Inhibitor Therapy Received

Variable	Patients, No. (%)			*P* value^a^
	Nivolumab (n = 51)	Pembrolizumab (n = 158)	Nivolumab-ipilimumab (n = 107)	
Age, median (IQR), y	66 (56-78)	69 (60-78)	53 (42-60)	.001
Sex				
Female	20 (39.2)	46 (29.1)	35 (32.7)	.40
Male	31 (60.8)	112 (70.9)	72 (67.3)	
Primary site				
Cutaneous	40 (78.4)	116 (73.4)	82 (76.6)	.60
Mucosal	3 (5.9)	15 (9.5)	7 (6.5)	
Unknown primary	8 (15.7)	27 (17.1)	18 (16.8)	
ECOG status				
0	19 (37.3)	61 (38.9)^b^	63 (58.9)	.001
1	27 (52.9)	63 (40.1)^b^	40 (37.4)	
2	4 (7.8)	29 (18.5)^b^	4 (3.7)	
3	1 (2.0)	4 (2.5)^b^	0	
Stage				
III	2 (3.9)	16 (10.1)	9 (8.4)	.006
IIIA	0	0	1 (0.9)	
IIIB	0	3 (1.9)	5 (4.7)	
IIIC	7 (13.7)	2 (1.3)	6 (5.6)	
IV	42 (82.4)	137 (86.7)	86 (80.4)	
Liver metastases				
Absent	37 (72.5)	118 (74.7)	76 (71.0)	.80
Present	14 (27.5)	40 (25.3)	31 (29.0)	
Brain metastases				
Absent	45 (88.2)	142 (89.9)	86 (80.4)	.08
Present	6 (11.8)	16 (10.1)	21 (19.6)	
*BRAF* V600E positive				
Absent	49 (96.1)	139 (88.0)	75 (70.1)	<.001
Present	2 (3.9)	19 (12.0)	32 (29.9)	
LDH level				
>ULN	31 (60.8)	85 (53.8)	61 (57.0)	.76
≤ULN	13 (25.5)	35 (22.2)	31 (29.0)	
Unknown	7 (13.7)	38 (24.1)	15 (14.0)	

Abbreviations: ECOG, Eastern Cooperative Oncology Group; LDH, lactate dehydrogenase; ULN, upper limit of normal.

^a^ *P* values for continuous variables are via nonparametric *t* test, and categorical variables are via χ^2^ test.

^b^ Missing 1 patient.

**Figure 1.**
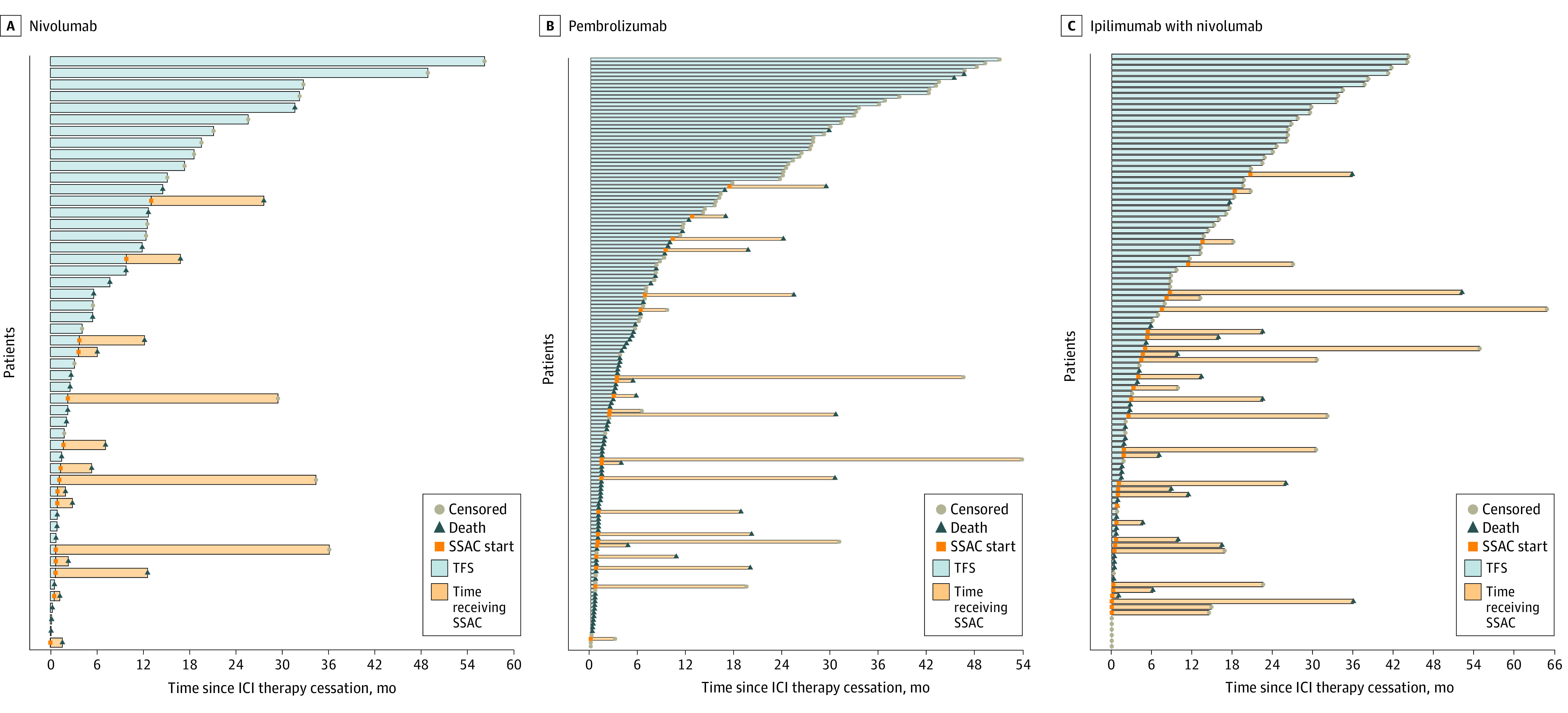
Swimmer Plots Demonstrating Patterns of Treatment-Free Survival (TFS) Periods After Cessation of Immune Checkpoint Inhibitor (ICI) Therapy for Patients With Advanced Melanoma The origin of the x-axis represents the date of ICI therapy discontinuation. The blue bar represents the TFS interval experienced by that patient. The presence of a square identifies the time of subsequent systemic anticancer (SSAC) therapy initiation, with the orange bar representing the time spent receiving that therapy regimen. A triangle represents death due to any cause, and a circle represents a censor at time last alive. Patients who continued receiving ICI therapy throughout the study period are censored at the origin.

To provide a descriptive analysis for the patterns of TFS seen among patients with advanced melanoma receiving first-line ICI therapy, swimmer plots for a representative selection of 50 patients from each ICI therapy subgroup are shown in Figure 1. The percentage of patients alive at 36 months after ICI therapy initiation was 33.3% (95% CI, 21.2%-52.1%) for the nivolumab cohort, 39.2% (95% CI, 31.3%-49.0%) for the pembrolizumab cohort, and 62.0% (95% CI, 52.5%-73.3%) for the nivolumab-ipilimumab cohort. Correspondingly, the restricted mean overall survival time, which represents the mean time spent alive over the 36-month period analyzed, was 21.2 months (95% CI, 17.4-25.1 months) for the nivolumab cohort, 21.3 months (95% CI, 19.0-23.7 months) for the pembrolizumab cohort, and 26.7 months (95% CI, 24.1-29.3 months) for the nivolumab-ipilimumab cohort. This finding suggests that patients receiving combined nivolumab-ipilimumab therapy had longer survival, on average, over the study period and were more likely to be alive at 36 months after therapy initiation compared with patients receiving anti–PD-1 monotherapy.

**Figure 2.**
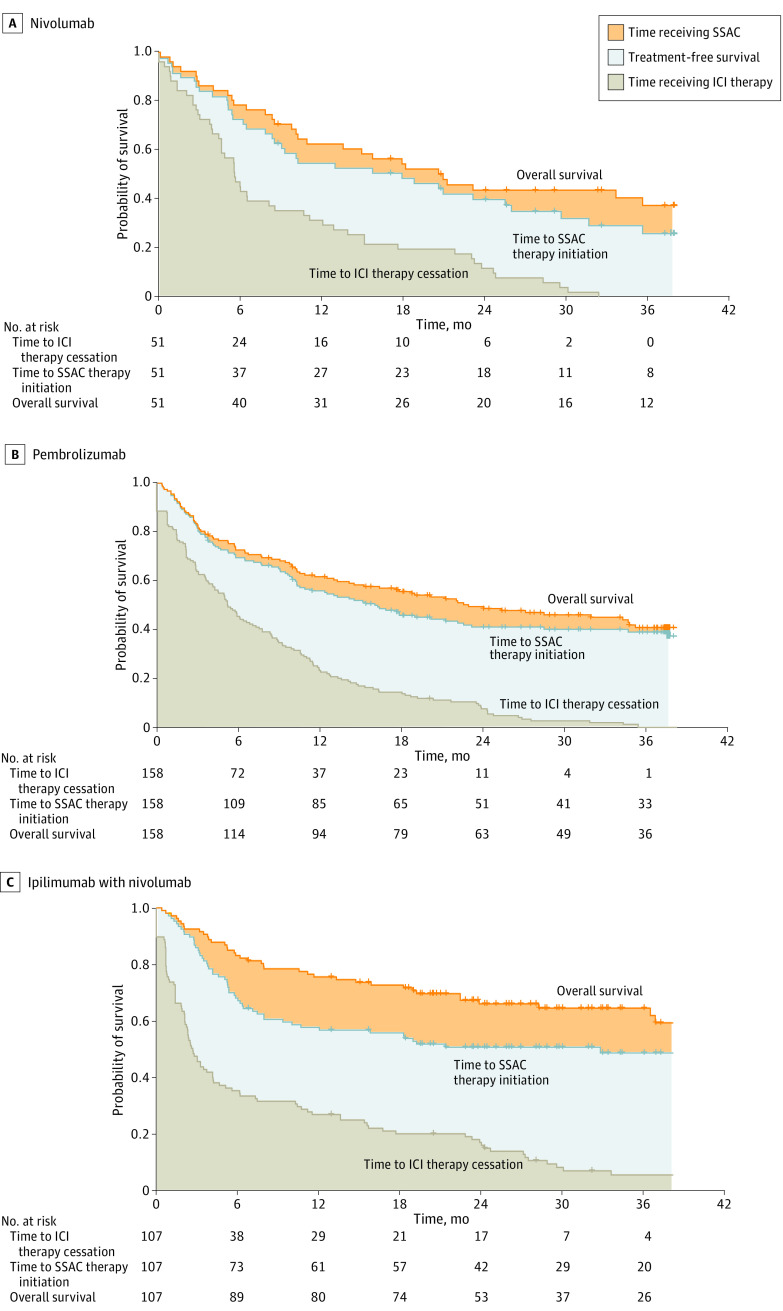
Treatment-Free Survival Over a 36-Month Period for Patients With Advanced Melanoma by Immune Checkpoint Inhibitor (ICI) Therapy Regimen Kaplan-Meier curves of the time to ICI therapy cessation, time to subsequent systemic anticancer (SSAC) therapy initiation, and overall survival are shown.

Patients who received single-agent nivolumab appeared to show a trend toward a longer period receiving ICI therapy (mean time, 9.8 months [95% CI, 7.3-12.3 months]) compared with those receiving single-agent pembrolizumab (mean time, 8.2 months [95% CI, 6.9-9.5 months]) or nivolumab-ipilimumab (mean time, 9.0 months [95% CI, 6.8-11.1 months]). Patients receiving nivolumab-ipilimumab spent a numerically longer period of the 36-month follow-up time alive and not receiving ICI therapy (mean time, 12.4 months [95% CI, 8.8-16.0 months]) compared with those receiving nivolumab (mean time, 8.9 months [95% CI, 4.4-13.5 months]) and pembrolizumab (mean time, 11.1 months [95% CI, 8.5-13.8 months]) (Figure 2). This corresponded to a total of 34.4%, 30.8%, and 24.7% of the 36-month landmark period spent free from any SSAC therapy for patients receiving combined nivolumab-ipilimumab, single-agent pembrolizumab, and single-agent nivolumab, respectively. Patients treated with combined nivolumab-ipilimumab also seemed to spend more time receiving subsequent SSAC therapy, accounting for a mean of 5.4 months (95% CI, 1.5-9.2 months) of the 36-month period compared with a mean of 2.5 months (95% CI, 0.9-7.9 months) for those receiving nivolumab and a mean of 2.0 months (95% CI, 0.6-5.3 months) for those receiving pembrolizumab. However, this difference may be associated with the larger number of *BRAF* V600E–positive patients in the combined nivolumab-ipilimumab group, as this subgroup experienced a numerically longer period receiving subsequent SSAC therapy (mean time, 8.9 months [95% CI, 1.9-16.0 months]) compared with those without this variant (mean time, 3.9 months [95% CI, 0.6-8.4 months]).

## Discussion

In this study, we present the first observational analysis of TFS outcomes for patients with advanced melanoma receiving first-line ICI therapy outside the clinical trial setting, to our knowledge. Our analysis found that patients receiving combination nivolumab-ipilimumab experienced improved overall survival, as well as a longer period free of SSAC therapy and death compared with patients receiving single-agent anti–PD-1 agents. These findings are in line with previous studies that have examined TFS in the advanced melanoma clinical trial setting.^9,10^

The longer TFS seen for patients receiving combination nivolumab-ipilimumab therapy may be associated with a more durable initial period of disease control, permitting SSAC therapy withdrawal, or the development of treatment-limiting adverse events followed by a treatment-free interval. These immune-related adverse events have been shown to be an established treatment biomarker of the likely benefit from ICI therapy.^13^ However, the subset of patients included in the combination nivolumab-ipilimumab cohort also had a lower ECOG status, were younger, and were more likely to be *BRAF* V600E variant positive, all of which may have been associated with the trend toward improved TFS in this group.

In addition, differences in baseline patient characteristics, as well as choice of second-line therapies, were likely associated with differences in the duration receiving SSAC therapy seen between groups. Specifically, the numerically longer time receiving SSAC therapies seen for the combination nivolumab-ipilimumab group may have been associated with the larger proportion of *BRAF* V600E–positive patients in this group, as this subgroup of patients has effective second-line therapy options. Moreover, a subset of patients receiving anti–PD-1 monotherapy may have been treated with second-line ipilimumab, which has a comparatively shorter duration of therapy, thus limiting their duration receiving SSAC therapy.

Taken in context, these findings suggest that combination nivolumab-ipilimumab may provide an additional benefit to patients with advanced melanoma by lengthening the time they spend not receiving SSAC therapy, which has been associated with improvements in quality-of-life outcomes. Moreover, this work emphasizes the importance of examining outcomes such as TFS to understand how time is spent by patients receiving different therapy regimens. Future studies investigating this outcome among patients with advanced melanoma may also use different study methods, such as a target trial approach, to further enhance the conclusions drawn.

### Limitations

This study has some limitations. It was retrospective and was restricted to patients who received therapy at centers in Alberta, Canada, which may limit the generalizability of the findings. In addition, given the observational nature of this study, differences in TFS between groups may also be partially associated with underlying differences in key clinical variables, such as ECOG status and patient age, as well as differences in tumor characteristics, such as the proportion of *BRAF* V600E–positive patients, which were not accounted for in this analysis. Furthermore, previous analyses^9,10^ had further stratified TFS based on the presence of immune-related adverse events into TFS with toxic effects and TFS without toxic effects, which could not be replicated in this cohort. Therefore, it is possible that, although patients receiving combination nivolumab-ipilimumab experienced longer TFS, a greater portion of this time was spent with additional toxic effects, as was seen in previous clinical trial analyses.^13^ Another limitation is the lack of information regarding choice of SSAC therapy for patients included in this analysis, which may have provided additional context for interpreting differences in duration receiving SSAC therapy seen in this study.

## Conclusions

In this cohort study of patients with advanced melanoma, those receiving first-line combined nivolumab-ipilimumab experienced a numerically longer TFS period compared with those receiving single-agent anti–PD-1 therapy. Additionally, TFS represented a patient-centric and informative end point.

## Notes

Supplement.
Data Sharing Statement
