# Supplementary material for: Treatment-Free Survival After Nivolumab vs Pembrolizumab vs Nivolumab-Ipilimumab for Advanced Melanoma
Source: JAMA Netw Open. 2023 Jun 23;6(6):e2319607. doi: 10.1001/jamanetworkopen.2023.19607 (PMC10290253; doi:10.1001/jamanetworkopen.2023.19607)
Supplement: Supplement. — Data Sharing Statement [file jamanetwopen-e2319607-s001.pdf]

Data Sharing Statement

Gupta. Treatment-Free Survival After Nivolumab vs Pembrolizumab vs Nivolumab-Ipilimumab for Advanced Melanoma. *JAMA Netw Open*. Published June 23, 2023. doi:10.1001/jamanetworkopen.2023.19607

Data
Data available: No

Additional Information
Explanation for why data not available: Data will be made available upon reasonable request of the corresponding author.
